# Ten-Year Survival after Liver Resection for Colorectal Metastases: Systematic Review and Meta-Analysis

**DOI:** 10.5402/2011/763245

**Published:** 2011-06-22

**Authors:** Saleh Abbas, Vincent Lam, Michael Hollands

**Affiliations:** ^1^Westmead Hospital, Wentworthville, NSW 2145, Australia; ^2^University of Western Sydney, Penrith, NSW 2751, Australia

## Abstract

*Background*. Liver resection in metastatic colorectal cancer is proved to result in five-year survival of 25–40%. Several factors have been investigated to look for prognostic factors stratifications such as resection margins, node involvement in the primary disease, and interval between the primary disease and liver metastases. *Methods*. We searched MEDLINE and EMBASE for studies that reported ten-year survival. Metaanalysis was performed to analyse the effect of recognised prognostic factors on cure rate for colorectal metastases. The meta-analysis was performed according to Ottawa-Newcastle method of analysis for nonrandomised trials and according to the guidelines of the PRISMA. *Results*. Eleven studies were included in the analysis, which showed a ten-year survival rate of 12–36%. Factors that have favourable impact are clear resection margin, low level of CEA, single metastatic deposit, and node negative disease. The only factor that excluded patients from cure is the positive status of the resection margin. *Conclusion*. Predicted ten-year survival after liver resection for colorectal metastases varies from 12 to 36%. Only positive resection margins resulted in no 10-year survivors. No patient can be excluded from consideration for liver resection so long the result is negative margins.

## 1. Introduction

It is known that 60–70% of recurrent colorectal cancer involves the liver and that the liver is the only involved organ in 35% of cases. Colorectal liver metastases used to be thought of as systemic disease that involves many organs and systems until recently when local liver therapy in the form of liver resection has been reported to results in 5-year survival of 27–39% and even longer survival and cure [1, 2]. Patients with liver metastases that are resectable but left untreated have average survival of 6–12 months and rarely longer than 2 years [3].

Over the last 20 years liver resection for colorectal metastases has seen many refinements; the improvement in anaesthesia and postoperative care have reduced the morbidity and mortality with subsequent more aggressive surgical approach. Strategies that have widened the indications for liver resection includes portal vein embolisation, staged liver resection, neoadjuvant chemotherapy, ablative procedures, and locoregional chemotherapy [4–7].

 Many studies have developed and validated scoring systems to predict prognosis and recurrence of colorectal metastases based on clinical and pathological data of large number of hepatic resections for colorectal metastases. These scores are based on variety of factors that include stage of the primary disease, time interval between diagnosis of the primary lesion and occurrence of liver metastases, level of CEA immediately prior to liver resection, size and number of resected liver lesions, surgical resection margin, blood transfusion, and bilateral distribution of liver disease [1, 8–10]. 

Various factors were found to have influence on disease recurrence and overall survival; resection margins and lymph nodes involvement are common predictors of recurrence. Other controversial factors are number and size of lesions, blood transfusion, and disease-free interval. Other factors are extrahepatic disease and portal nodes metastases [11–14].

This systematic review is conducted to evaluate the risk factors influence of overall long-term survival following hepatic resection for colorectal metastases.

## 2. Methods

### 2.1. Search and Study Identification

Electronic search was performed and relevant reports were identified using electronic databases (MEDLINE 1950–2010 and EMBASE 1980–2010), the search was restricted to human adults and English language literature. The search terms used were colorectal neoplasm, overall survival, and disease-free survival. All the references used in the published original and review studies were searched to identify more studies.

### 2.2. Criteria for Study Selection

To be included, studies had to meet the following criteria.

1. Design: prospective or retrospective cohort studies.
2. Population: patients with liver metastases from colorectal cancer who had liver resection as a curative treatment.
3. Exposure: surgical liver resection for metastases whether anatomic resection or segmental nonanatomic resection regardless of whether they had or did not have adjuvant chemotherapy. That included studies that evaluated patients' survival following re-resection following primary liver resection.
4. Outcome: overall ten-year survival following liver resection for colorectal liver metastases.

Duplicate publications were excluded and wherever publications that evaluated the same population group were encountered, the report with the most relevant and comprehensive data was selected.

### 2.3. Data Extraction

Articles that met all the inclusion criteria were retrieved as full text articles. Two independent reviewers using standard data collection form extracted all relevant data from the full text articles. Inconsistencies were resolved by discussion to reach a reasonable consensus. Whenever missing data were encountered, the authors were contacted to request the data required to be included in the meta-analysis. One study in non-English language was encountered and was excluded.

### 2.4. Quality Assessment

Methodological quality of the studies was evaluated independently by two reviewers using the Newcastle-Ottawa Scale [15]. A quality score was calculated on the basis of the following components: selection of the study groups (0–4 points), quality of the adjustment for confounding variables (0–2), and outcome of interest in the study population (0–3 points). A higher score represents better methodological quality.

### 2.5. Statistical Analysis

Odds ratio (OR) of overall survival was used as the primary effect estimate in this meta-analysis. From the eligible studies that met the inclusion criteria, estimates of the OR and its associated 95% confidence interval (CI) were calculated using the Review Manager software (Version 5 for Windows, Copenhagen, Denmark; The Nordic Cochrane Centre, The Cochrane Collaboration, 2008). Data that could not be extracted directly were reconstructed indirectly by two reviewers when required. 

Prespecified factors that was thought to affect the overall survival after liver resection for colorectal metastases were analysed. Those included resection margin, tumour size, number of metastases, bilateral versus unilateral disease, T stage of the primary, lymph nodes positive primary versus lymph nodes negative, disease-free interval, CEA level, and blood transfusion. Sensitivity analysis on the included studies was conducted on the Review Manager.

Heterogeneity between the included studies was appraised using the *Q* measure for statistical significance and the *I^2^* measure for the amount of heterogeneity, with *P* < .1 being statistically significant and *I^2^* > 25% showing important heterogeneity. A random effect model based on DerSimonian-Laird estimator was used wherever there was significant heterogeneity, and fixed effect model based on Mantel-Haenszel estimator was used when there was no significant heterogeneity [16]. We conducted Begg's test and the Harbord modified test to identify publication bias for small study effect, with *P* > .5 being statistically significant [17]. The results of this systematic review were reported using the Preferred Reporting Items for Systematic Reviews and Meta-Analyses (PRISMA) guidelines [18].

## 3. Results

### 3.1. Study Characteristics and Methodological Quality

**Figure 1:**
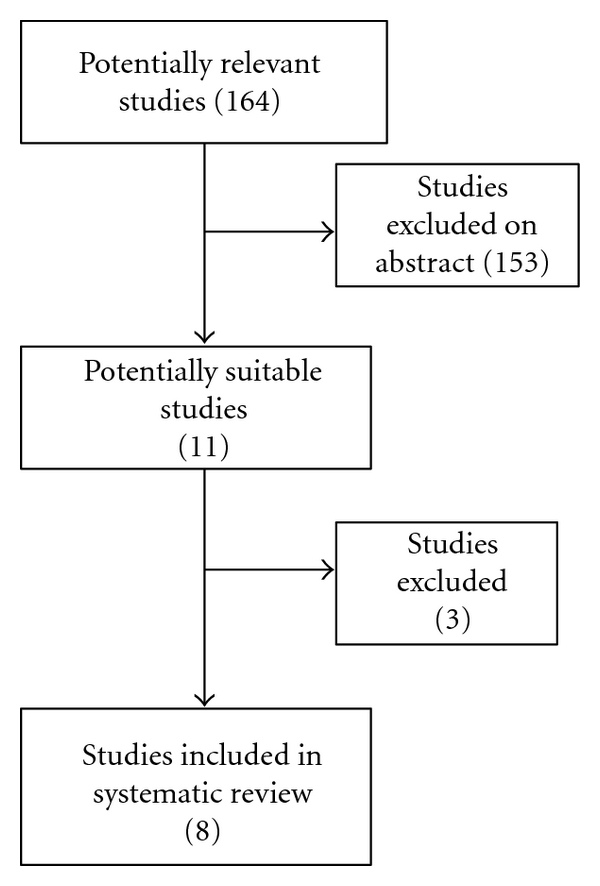
Flow diagram of study selection process.

The initial search revealed 164 titles, abstracts for those articles (Figure 1) were reviewed, 28 articles were considered to be potentially useful for inclusion, and their full text was retrieved and reviewed. Seventeen of these 28 articles were subsequently excluded from the meta-analysis as they did not meet the inclusion criteria. Eleven original reports (all retrospective cohort studies) had enough data to investigate the role of different variables on overall long-term survival after liver resection for colorectal metastases. Two studies were excluded due to the fact that they were a duplication of the same study population [19, 20]. One further study was excluded because it had no data that can be used in the meta-analysis [21]. The included studies were published between 1995 and 2009 (Table 1). The methodological quality of the included studies is shown in (Table 2). There was no statistical evidence of publication bias between the included studies.

There was no statistical evidence of publication bias among the included studies based on the funnel plot used in Review Manager.

### 3.2. Patients Characteristics

The eleven studies reviewed 3442 patients who had liver resection for colorectal metastases, the eight included studies had a total number of 2387 patients, all studies reported five- and ten-year survival [22–29]. Overall 5-year survival was 21–51% and overall ten-year survival was 12–36%. These studies had variably reported the impact of different factors on overall survival; these factors included resection margins, size of the largest liver lesion, number of liver lesions, distribution of lesions, CEA levels prior to liver resection, lymph node status of the primary, satellite configuration of liver metastases, type of resection, extrahepatic disease, and whether the liver metastases were presented in a synchronous or metachronous to the diagnosis of the primary colorectal cancer.

### 3.3. Disease-Free Survival

In the study of Tomlinson et al. [28] they reported that 34% of patients who are disease-free at 5 years after hepatic resection did experience recurrence. This figure is high compared with other reports by Minagawa et al., Giuliante et al., and Scheele et al. who reported 50- and 10-year disease-free survival of 26 and 23%, 28.2 and 25.4%, and 33.6 and 25.4%, respectively [22, 25, 26]. From these data we conclude that 5-year survival does not equate cure. Since recurrence after 10-year of disease-free survival is rare; ten-year survival could be considered as definitive cure.

Factors that influence disease-free survival and may predict that has been reported only in the study by Minagawa et al. [25], hence it was not suitable to conduct meta-analysis on this category of outcome.

### 3.4. Influence of Resection Margins

**Figure 2:**
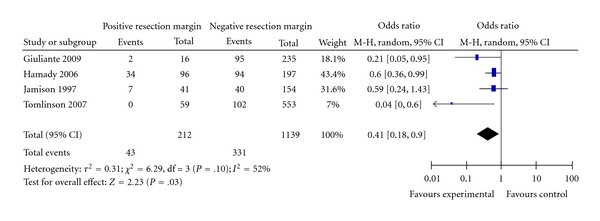
Positive and negative margins.

 Four studies reported adequate data to determine the relationship between positive and negative resection margin and long-term survival. The average overall survival for positive margin was 29%, which is significantly better compared with 20% of negative resection margin (*P* = .03) with odds ratio of 0.41 (95% CI 0.18–0.9). There was moderate degree of heterogeneity *I^2^* 52%; however, this heterogeneity was eliminated when prespecified sensitivity analysis was performed by elimination of the study by Tomlinson et al. [28] that had a wide 95% CI and that has little overlap with other studies. The result of the meta-analysis after this elimination showed OR of 0.55 (0.95% CI 0.36–0.84) with *P* = .005, which ensures that the results of the meta-analysis are robust (Figure 2). 

**Figure 3:**
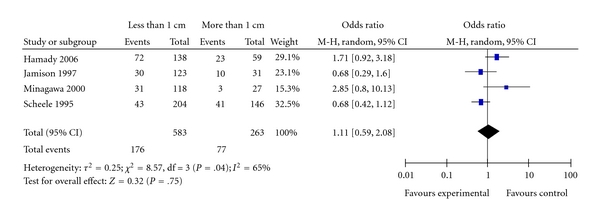
Resection margin less than 1 cm or more than 1 cm.

Four studies reported the influence of wider negative margin of more than 1cm compared with negative margin of 0–10 mm [23–26]. Wider resection margin had no beneficial effect on overall survival. Pooled analysis for the likelihood of survival is shown in Figure 3 (OR = 1.11; 95% CI: 0.59–2.08; *P* = .75). Moderate heterogeneity was seen. The pooled estimate was robust: omission of individual study at a time did not change the statistical results (data not shown).

**Figure 4:**
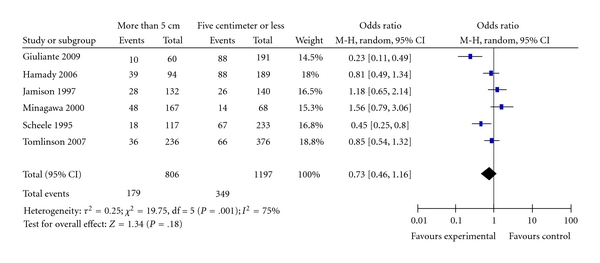
Tumour size.

Six studies reported the effect of tumour size on overall survival [22–26, 28]; meta-analysis of the studies that reported analysis data on patients liver metastases more than 5 cm compared with patients who had liver lesions of 5 cm or less, showed no prognostic relationship between size of the resected tumour and overall patients' survival.  Figure 4 shows the results of pooled estimate for survival in relation to tumour size (OR = 0.73, 95% CI: 0.46–1.16; *P* = .18). There was moderate heterogeneity among the included studies *I^2^*  = 75%, the results were robust, omission of the study by Giuliante et al. [22] resulted in very close odds ratio and removed the heterogeneity.

**Figure 5:**
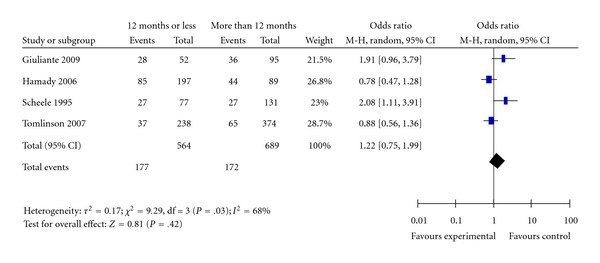
Disease-free interval.

Four studies reported data on the effect of time interval between the diagnoses of the primary colorectal cancer and the occurrence of liver metastases on the overall patients' survival. Pooled estimate of the survival time after liver resection for colorectal metastases showed no significant prognostic relationship (OR = 1.22; 95% CI: 0.75–1.99; *P* = .42) Figure 5. There was moderate degree of heterogeneity (*I^2^*  = 68%). The analysis results were robust, exclusion of the study by Scheele et al. [26] removed the heterogeneity and the results and resulted in OR = 1.03; 95% CI: 0.64–1.65, data not shown.

**Figure 6:**
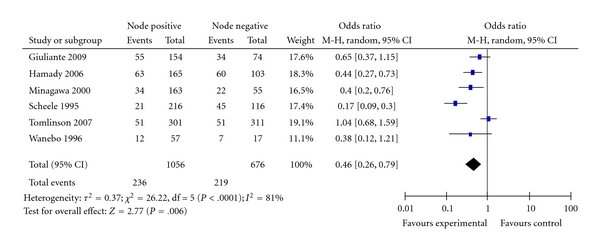
Nodal disease of the primary cancer.

Six studies reported data to determine the relationship between lymph nodes metastasis status of the primary colorectal cancer and survival after liver resection for colorectal metastases [22, 23, 25, 26, 28, 29].  Figure 6 shows the forest plot with pooled estimates of the odds ratio of survival in patients who had node positive primary disease compared with those who had node negative disease. The overall ten-year survival for node negative disease was 32% compared with 22% for nodes positive primary disease (OR = 0.46; 95% CI: 0.26–0.79; *P* = .006), the pooled analysis showed significant heterogeneity *I^2^*  = 81%; omission of the study by Tomlinson et al. [28] reduced the heterogeneity to insignificant level with results of odds ratio of 0.38; 95% CI: 0.23–6; *P* < .0001, which ensures the robustness of the pooled estimates of the effect.

**Figure 7:**
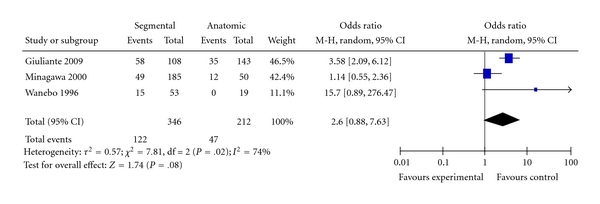
Type of resection.

Three studies reported data for determination of the relationship between types of liver resection (segmental or anatomic) [22, 25, 29].  Figure 7 shows the Forest plot of the pooled estimates of long-term survival for patients who had segmental resection compared with patients who had anatomic resection (right hepatectomy, left hepatectomy, or trisectionectomy). The type of resection did not have any significant impact on overall survival (OR = 2.60; 95% CI: 0.88–7.63; *P* = .08), there was significant heterogeneity among the included studies. Omission of individual studies did not change the results of the analysis.

### 3.5. CEA Levels

**Figure 8:**
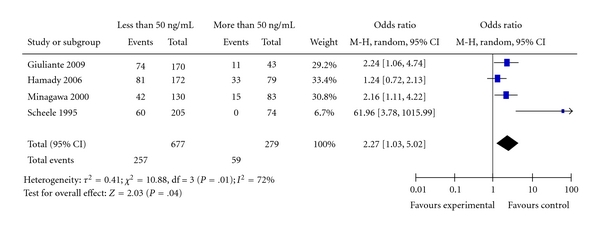
CEA level.

Four studies provided data for determining the relationship of CEA levels, prior to resection of colorectal liver metastases, and the overall patients' survival [22, 23, 25, 26].  Figure 8 shows the Forest plot with the pooled estimate for likelihood of ten-year survival for patients with CEA levels more than 50 ng/mL and those with CEA levels less than 50 ng/mL. Overall pooled survival for those with CEA level less than 50 ng/mL is 37% compared with 19% for those with CEA greater than 50 ng/mL. The results show statistically significant better survival in those with low CEA levels (OR = 2.27; 95% CI: 1.03–5.02; *P* = .04). There is significant heterogeneity (*I^2^*  = 72%); omission of the study by Scheele et al. [26], which looks like an outlier has removed the heterogeneity, and still the results were significant (OR = 1.71; 95% CI: 1.15–2.55; *P* = .009; data not shown), which confirms the robust results of the analysis. 

### 3.6. Distribution of Liver Lesions

**Figure 9:**
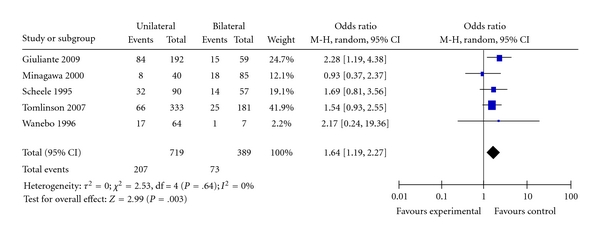
Distribution of liver lesions.

Five studies provided data to determine the effect of bilateral distribution of resected colorectal metastases on the ten-year survival [22, 25, 26, 28, 29]. Ten-year survival was 36% compared with 18% in patients who had bilateral disease resected.  Figure 9 shows the Forest plot of the pooled estimate for ten-year survival, the presence of bilateral disease in the liver leads to significantly reduced long-term survival (OR = 1.64; 95% CI: 1.19–2.27; *P* = .003. There was no significant heterogeneity among the included studies *I^2^*  = 0%.

### 3.7. Number of Liver Lesions

**Figure 10:**
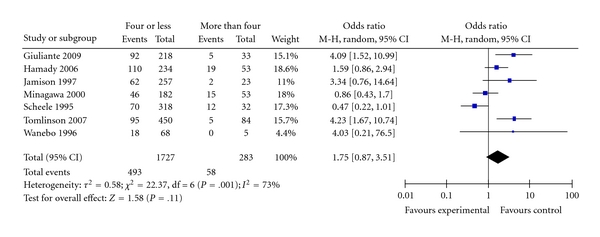
Number of liver lesions.

Seven studies reported data to determine the effect of the number of the resected liver lesions on long-term survival [22–26, 28, 29]; these studies compared survival in patients with four or less lesions versus patients who had more than four lesions and found that ten-year survival for patients with four or less lesion was 38% compared with 20% of those who had more than four lesions (OR = 1.75; 95% CI: 0.87–3.51; *P* = .11); there was a significant heterogeneity *I^2^*  = 73%; omission of individual studies made no statistical difference; however, omission of three studies, Scheele et al., Minagawa et al., and Tomlinson et al. [25, 26, 28] removes the heterogeneity and results in significantly better survival in patients who had four or less lesions (OR = 2.26; 95% CI: 1.36–3.75; *P* = .002), data not shown. This made it not possible to make any valid conclusion about the effect of lesion's number on overall survival (Figure 10).

### 3.8. Synchronous versus Metachronous Metastases

**Figure 11:**
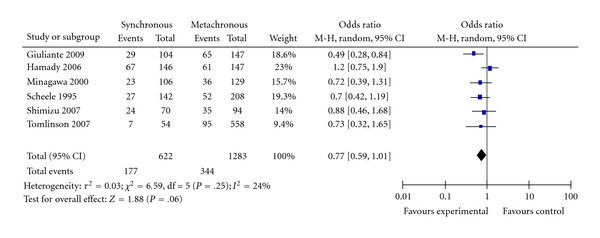
Synchronous versus metachronous metastases.

Six studies provided data to determine the relationship of timing of liver metastases; whether it was found at the time of diagnosis of the primary colorectal cancer or afterward [22, 23, 25–28].  Figure 11 shows the Forest plot with pooled estimate of likelihood of survival for patients who had liver metastases diagnosed synchronous with the primary colorectal cancer compared with those who developed liver metastases afterward (metachronous; OR = 0.77; 95% CI: 0.59–1.01; *P* = .06). The timing of liver metastases has no significant effect on long-term survival. 

### 3.9. Effect of Blood Transfusion

**Figure 12:**
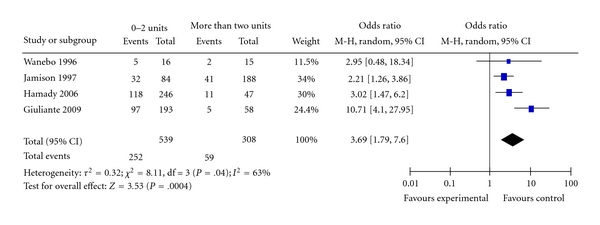
Blood transfusion.

Four studies provided data for determining the relationship of blood transfusion, after liver resection, and long-term survival [22–24, 29].  Figure 12 shows the Forest plot with the pooled estimate for long-term survival. Patients who received two or less units had significantly better survival than patients who had more than two units (OR = 3.69; CI: 1.79–7.60; *P* = .0004). There was significant heterogeneity among the included studies *I^2^*  = 63%. Omission of the study of Giuliante et al. [22] removed the heterogeneity, and the pooled estimate remained valid (OR = 2.51; CI: 1.63–3.85; *P* < .0001), data not shown.

**Figure 13:**
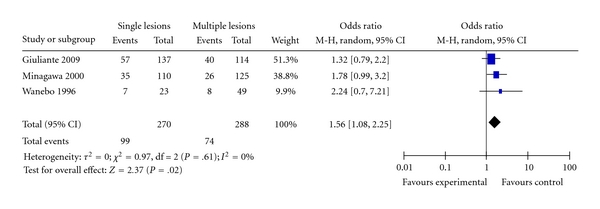
Single versus multiple lesions.

Three studies provided data that compared the difference in survival between patients who had a single lesion resected and those who had more than a single lesion [22, 25, 29].  Figure 13 shows the Forest plot of the pooled estimates of survival for patients with single lesion compared to patients who had multiple lesions. Patients with a single lesions had significantly better prognosis than those with multiple lesions (OR = 1.56; 95% CI: 1.08–2.25; *P* = .02).

**Figure 14:**
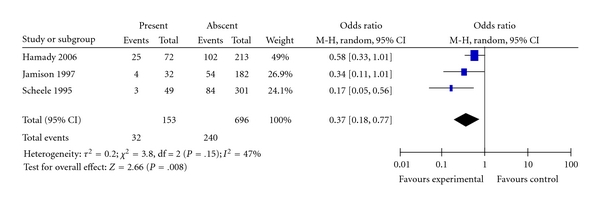
Satellite lesions.

Three studies provided data that compared survival of patients who had satellite lesions along with larger lesion or lesions compared with those who had no satellite lesions [23, 24, 26].  Figure 14 shows the Forest plot of the pooled estimate of survival, the estimate shows significantly better survival for patients who had no satellite lesions compared with those who had satellite lesions (OR = 0.37; CI: 0.18–0.77; *P* = .008), there was significant heterogeneity among the included studies.

## 4. Discussion

This systematic review and meta-analysis showed that factors that affect long-term survival following hepatic resection, for colorectal cancer metastases, include clear resection margins, advanced primary colorectal cancer with nodal metastases, CEA levels, distribution of liver lesions, timing of diagnosis of liver metastases (synchronous or metachronous), quantity of blood transfusion, single lesion compared with multiple lesions, and presence or absence of satellite nodules close to the main lesion. Patients who had clear resection margins had significantly better long-term survival than those with positive resection margins. Patients who had early stage colorectal cancer with no lymph nodes metastases had better survival than those with lymph nodes metastases. It also showed that metachronous presentation of liver metastases is a good prognostic factor compared with synchronous presentation. A single liver lesion particularly in the absence of satellite nodules has better outcome than multiple lesions with or without satellite nodules; however, when comparison was made between patients with more than four nodules and those with four or less lesions, there was no difference in overall long-term survival. CEA levels less than 50 ng/mL, unilateral liver disease, and two units or less of perioperative blood transfusion were found to be favourable prognostic factors. There was no single prognostic factor of sufficient power to predict long-term survival and cure.

Other factors that were analysed in this meta-analysis and found not to have significant influence on long-term survival included a the width of resection margin, it was found that if the resection margin is clear there is no survival benefit from a wider resection margin more than 1 cm. Whether the resected largest lesion was more or less than 5 cm did not have effect on survival. The interval between the diagnosis of the primary and liver recurrence less or more than 12 months did not seem to affect long term survival. Whether the lesion removed in anatomic resection technique or segmental resection, number of resected lesions, and synchronous versus metachronous metastases, all had no effect on long-term survival.

During the past two decades, liver resection for colorectal liver metastases has been increasing the standard of care whenever the disease is limited to the liver and is technically possible by leaving adequate liver remnants. There is overwhelming evidence to support the survival benefit with reports of actuarial 5-year survival of 25–40% compared with patients who are treated only with chemotherapy who rarely survive up to five years [30, 31]. With technical advances and improved perioperative the mortality of liver resection is less than 5%, Wei et al. reported 1.7% mortality in a large series with morbidity of 19% [21]. This improvement makes liver resection a standard treatment for colorectal metastases [12, 20, 26, 28].

Tomlinson et al. had investigated risk factors for 10-year survival and redesigned the original score devised by the same investigators. The original score had five components with one point for each component that includes tumour number more than four, size more than 5 cm, CEA level more than 200 ng/mL, and disease-free interval less than 12 months and positive resection margin with one point for each. After analysing 10-year survival, patients fell in two groups; the low risk group (0–2 points) who had 10-year survival of 21% and high-risk group (3–5 points) with 10-year survival of 10% (*P* < .0001) [1, 28]. No patients with positive resection margin had survived for 10 years, which seems to be the only factor that ruled out any possibility of cure [28]. This raises the question of the benefit of neoadjuvant chemotherapy particularly now there are more effective agents that may increase the rate of complete resection, the effect of that approach on survival is not known. 

It was not possible in this review to analyse the effect of adjuvant chemotherapy on survival after hepatic resection for colorectal metastases. However, most of the study patients had been treated in the era when the standard chemotherapy was 5-flourouracil, which has limited effect, compared with the modern chemotherapeutic agents like irinotecan, oxaliplatin, and bevacizumab [28]. This makes the evaluation of the pure curative benefit from surgical resection relatively difficult to assess.

The improvement of outcome in liver resection has been attributed to a combination of factors such as aggressive surgical treatment, improved chemotherapy, and improvement of preoperative imaging and patient selection [32–34].

Repeat hepatectomy for recurrent hepatic disease following initial liver resection is being increasingly used, also staged liver resection and resection of isolated extrahepatic disease is being more and more utilised with encouraging results that lead to improvement in survival [4, 34–37]. Despite all the advances and improvements, it remains not possible to discriminate with reasonable certainty which subset of patients is likely to be cured or live more than 5 years. Repeat liver resection gives overall survival results of up to 52% in one series and is comparable to the results of initial liver resection [37].

Improvement of preoperative staging by liberal use of helical CT scans and MRI and the introduction of PET scan and PET CT have allowed for better patients selection by early detection of extrahepatic and bilobar disease [33, 38–40].

Many authors have reported using radiofrequency ablation in conjunction with surgery for nonresectable liver metastases either intraoperative or postoperative with variable results [21, 32], the impact of this technique on overall long-term survival remains questionable.

The emergence of new chemotherapeutic agents such as oxaliplatin, irinotecan, and bevacizumab has increased the treatment option available for clinicians to deal with metastatic colorectal disease. These new agents have been used in conjunction with liver resection either as neoadjuvant or adjuvant manner in many studies but the effect of this approach on survival has not been test in a randomised controlled trial [5, 41, 42]. Chemotherapy is being commonly used as an adjuvant agents following liver resection particularly in patients who had no previous chemotherapy, the survival benefit of this approach remains to be proved [21]. Another potential use is to downstage potentially resectable liver metastases, in this situation patients with advanced disease are offered resection if they show good response to chemotherapy, this strategy has been used with limited success [5, 34].

In conclusion this review defines 10-year survival and cure to be between 12% and 28%; we described the factors that affect survival in this meta-analysis. There is no single factor that was of sufficient power to rule out cure with the possible exception of positive resection margin. This leads to the fact that patients' selection for resection with disease limited to the liver or liver disease with resectable extrahepatic metastases remains a matter of trial and error particularly for patients who have marginal suitability for resection. This review also indicates that we need newer prognostic factors perhaps based on tumour biology that may discriminate between curable and noncurable metastatic colorectal cancer. An important limitation of this study that reflects the quality of the available data is the fact that raw data was not available to all the studies' patients and the presence of heterogeneity among the included studies and the fact that those studies are generally retrospective reviews. Also patients who had 10-year survival are likely to have been treated prior to the era of PET scan routine use and likely have been treated with old and less effective chemotherapeutic agents.

## Figures and Tables

**Table 1:** Characteristics of the included studies.

Study	Study design	Total number of patients	5-year survival	10-year survival
Giuliante et al. 2009 [22]	Retrospective	251	38.9%	24.2%
Hamady et al. 2006 [23]	Retrospective	293	44%	36%
Jamison et al. 1997 [24]	Retrospective	280	27%	20%
Minagawa et al. 2000 [25]	Retrospective	235	38%	26%
Scheele et al. 1995 [26]	Retrospective	469	39.3%	23.6%
Shimizu et al. 2007 [27]	Retrospective	164	51.8%	36.6%
Tomlinson et al. 2007 [28]	Retrospective	612	21%	17%
Wanebo et al. 1996 [29]	Retrospective	74	24%	12%

**Table 2:** Quality assessment of included studies (Newcastle-Ottawa Scale).

Study	Selection	Comparability	Outcome
Giuliante et al. 2009 [22]	4	1	3
Hamady et al. 2006 [23]	4	2	3
Jamison et al. 1997 [24]	4	2	3
Minagawa et al. 2000 [25]	4	1	3
Scheele et al. 1995 [26]	4	2	3
Shimizu et al. 2007 [27]	4	1	3
Tomlinson et al. 2007 [28]	4	2	3
Wanebo et al. 1996 [29]	4	2	3

## References

[1] Fong Y, Fortner J, Sun RL, Brennan MF, Blumgart LH (1999). Clinical score for predicting recurrence after hepatic resection for metastatic colorectal cancer: analysis of 1001 consecutive cases. *Annals of Surgery*.

[2] Choti MA, Sitzmann JV, Tiburi MF (2002). Trends in long-term survival following liver resection for hepatic colorectal metastases. *Annals of Surgery*.

[3] Wagner JS, Adson MA, Van Heerden JA (1984). The natural history of hepatic metastases from colorectal cancer. A comparison with resective treatment. *Annals of Surgery*.

[4] Petrowsky H, Gonen M, Jarnagin W (2002). Second liver resections are safe and effective treatment for recurrent hepatic metastases from colorectal cancer: a Bi-institutional analysis. *Annals of Surgery*.

[5] Mackay HJ, Billingsley K, Gallinger S (2005). A multicenter phase II study of “adjuvant” irinotecan following resection of colorectal hepatic metastases. *American Journal of Clinical Oncology*.

[6] Kemeny N, Huang Y, Cohen AM (1999). Hepatic arterial infusion of chemotherapy after resection of hepatic metastases from colorectal cancer. *The New England Journal of Medicine*.

[7] Tanaka K, Adam R, Shimada H, Azoulay D, Lévi F, Bismuth H (2003). Role of neoadjuvant chemotherapy in the treatment of multiple colorectal metastases to the liver. *British Journal of Surgery*.

[8] Minagawa M, Yamamoto J, Kosuge T, Matsuyama Y, Miyagawa SI, Makuuchi M (2007). Simplified staging system for predicting the prognosis of patients with resectable liver metastasis: development and validation. *Archives of Surgery*.

[9] Zakaria S, Donohue JH, Que FG (2007). Hepatic resection for colorectal metastases: Value for risk scoring systems?. *Annals of Surgery*.

[10] Nordlinger B, Guiguet M, Vaillant JC (1996). Surgical resection of colorectal carcinoma metastases to the liver: a prognostic scoring system to improve case selection, based on 1568 patients. *Cancer*.

[11] Mann CD, Metcalfe MS, Leopardi LN, Maddern GJ (2004). The clinical risk score: Emerging as a reliable preoperative prognostic index in hepatectomy for colorectal metastases. *Archives of Surgery*.

[12] Gayowski TJ, Iwatsuki S, Madariaga JR (1994). Experience in hepatic resection for metastatic colorectal cancer: analysis of clinical and pathologic risk factors. *Surgery*.

[13] Jaeck D, Nakano H, Bachellier P (2002). Significance of hepatic pedicle lymph node involvement in patients with colorectal liver metastases: a prospective study. *Annals of Surgical Oncology*.

[14] Kato T, Yasui K, Hirai T (2003). Therapeutic results for hepatic metastasis of colorectal cancer with special reference to effectiveness of hepatectomy: analysis of prognostic factors for 763 cases recorded at 18 institutions. *Diseases of the Colon and Rectum*.

[15] Wells GA, Shea B, O'Connell D, Peterson J, Welch V The Newcastle-Ottawa Scale (NOS) for assessing the Quality of Nonrandomised Studies in Metaanalyses. http://www.ohri.ca/programs/clinicalepidemiology/oxford.htm.

[16] Higgins JPT, Green S (2006). *Cocharne Handbook for Systematic Reviews of Intervention*.

[17] Dickersin K, Rothstein HR, Sutton AJ, Borenstein M (2005). Publication bias: recognizing the problem, understanding its origins and scope, and preventing harm. *Publication Biasin Meta-Analysis; Prevention, Assessment and Adjustments*.

[18] Moher D, Liberati A, Tetzlaff J (2009). Preferred reporting items for systematic reviews and meta-analyses: the PRISMA statement. *PLoS Medicine*.

[19] Nuzzo G, Giuliante F, Ardito F (2008). Influence of surgical margin on type of recurrence after liver resection for colorectal metastases: a single-center experience. *Surgery*.

[20] D'Angelica M, Brennan MF, Fortner JG, Cohen AM, Blumgart LH, Fong Y (1997). Ninety-six five-year survivors after liver Resection for metastatic colorectal cancer. *Journal of the American College of Surgeons*.

[21] Wei AC, Greig PD, Grant D, Taylor B, Langer B, Gallinger S (2006). Survival after hepatic resection for colorectal metastases: a 10-year experience. *Annals of Surgical Oncology*.

[22] Giuliante F, Ardito F, Vellone M (2009). Role of the surgeon as a variable in long-term survival after liver resection for colorectal metastases. *Journal of Surgical Oncology*.

[23] Hamady ZZR, Cameron IC, Wyatt J, Prasad RK, Toogood GJ, Lodge JPA (2006). Resection margin in patients undergoing hepatectomy for colorectal liver metastasis: a critical appraisal of the 1 cm rule. *European Journal of Surgical Oncology*.

[24] Jamison RL, Donohue JH, Nagorney DM, Rosen CB, Harmsen WS, Ilstrup DM (1997). Hepatic resection for metastatic colorectal cancer results in cure for some patients. *Archives of Surgery*.

[25] Minagawa M, Makuuchi M, Torzilli G (2000). Extension of the frontiers of surgical indications in the treatment of liver metastases from colorectal cancer: long-term results. *Annals of Surgery*.

[26] Scheele J, Stang R, Altendorf-Hofmann A, Paul M (1995). Resection of colorectal liver metastases. *World Journal of Surgery*.

[27] Shimizu Y, Yasui K, Sano T (2007). Treatment strategy for synchronous metastases of colorectal cancer: is hepatic resection after an observation interval appropriate?. *Langenbeck's Archives of Surgery*.

[28] Tomlinson JS, Jarnagin WR, DeMatteo RP (2007). Actual 10-year survival after resection of colorectal liver metastases defines cure. *Journal of Clinical Oncology*.

[29] Wanebo HJ, Chu QD, Vezeridis MP, Soderberg C (1996). Patient selection for hepatic resection of colorectal metastases. *Archives of Surgery*.

[30] Hwitzhur HF, Novotony W (2004). Bevacizumab plus irinotecan, flourouracil, and leucovorine for metastatic colorectal cancer. *The New England Journal of Medicine*.

[31] Martin A (1987). Resection of liver metastases: When is it worthwhile?. *World Journal of Surgery*.

[32] Abdalla EK, Vauthey JN, Ellis LM (2004). Recurrence and outcomes following hepatic resection, radiofrequency ablation, and combined resection/ablation for colorectal liver metastases. *Annals of Surgery*.

[33] Truant S, Huglo D, Hebbar M, Ernst O, Steinling M, Pruvot FR (2005). Prospective evaluation of the impact of [^18^F]fluoro-2-deoxy-D- glucose positron emission tomography of resectable colorectal liver metastases. *British Journal of Surgery*.

[34] Elias D, Baton O, Sideris L (2005). Hepatectomy plus intraoperative radiofrequency ablation and chemotherapy to treat technically unresectable multiple colorectal liver metastases. *Journal of Surgical Oncology*.

[35] Adam R, Laurent A, Azoulay D (2000). Two-stage hepatectomy: a planned strategy to treat irresectable liver tumors. *Annals of Surgery*.

[36] Roh MS (2004). Expanding the indications for hepatic resection in patients with colorectal liver metastases. *Annals of Surgical Oncology*.

[37] Shah SA, Haddad R, Al-Sukhni W (2006). Surgical resection of hepatic and pulmonary metastases from colorectal carcinoma. *Journal of the American College of Surgeons*.

[38] Valls C, Andía E, Sánchez A (2001). Hepatic metastases from colorectal cancer: preoperative detection and assessment of resectability with helical CT. *Radiology*.

[39] Valls C, Lopez E, Gumà A (1998). Helical CT versus CT arterial portography in the detection of hepatic metastasis of colorectal carcinoma. *American Journal of Roentgenology*.

[40] Fernandez FG, Drebin JA, Linehan DC (2004). Five-year survival after resection of hepatic metastases from colorectal cancer in patients screened by positron emission tomography with F-18 fluorodeoxyglucose (FDG-PET). *Annals of Surgery*.

[41] de Gramont A, Figer A, Seymour M (2000). Leucovorin and fluorouracil with or without oxaliplatin as first-line treatment in advanced colorectal cancer. *Journal of Clinical Oncology*.

[42] Saltz LB, Cox JV, Blanke C (2000). Irinotecan plus fluorouracil and leucovorin for metastatic colorectal cancer. *The New England Journal of Medicine*.

